# Unusual Structural Features in the Adduct of Dirhodium Tetraacetate with Lysozyme

**DOI:** 10.3390/ijms22031496

**Published:** 2021-02-02

**Authors:** Domenico Loreto, Giarita Ferraro, Antonello Merlino

**Affiliations:** 1 Department of Chemical Sciences, University of Naples Federico II, 80126 Napoli, Campania, Italy; domenico.loreto@unina.it; 2 Department of Chemistry “Ugo Schiff”, University of Florence, via della Lastruccia, 3-13, 50019 Sesto Fiorentino, Florence, Italy; giarita.ferraro@unifi.it

**Keywords:** Rh compounds, metal complexes, metallodrugs, protein metalation, dirhodium carboxylates

## Abstract

The structures of the adducts formed upon reaction of the cytotoxic paddlewheel dirhodium complex [Rh_2_(μ-O_2_CCH_3_)_4_] with the model protein hen egg white lysozyme (HEWL) under different experimental conditions are reported. Results indicate that [Rh_2_(μ-O_2_CCH_3_)_4_] extensively reacts with HEWL:it in part breaks down, at variance with what happens in reactions with other proteins. A Rh center coordinates the side chains of Arg14 and His15. Dimeric Rh–Rh units with Rh–Rh distances between 2.3 and 2.5 Å are bound to the side chains of Asp18, Asp101, Asn93, and Lys96, while a dirhodium unit with a Rh–Rh distance of 3.2–3.4 Å binds the C-terminal carboxylate and the side chain of Lys13 at the interface between two symmetry-related molecules. An additional monometallic fragment binds the side chain of Lys33. These data, which are supported by replicated structural determinations, shed light on the reactivity of dirhodium tetracarboxylates with proteins, providing useful information for the design of new Rh-containing biomaterials with an array of potential applications in the field of catalysis or of medicinal chemistry and valuable insight into the mechanism of action of these potential anticancer agents.

## 1. Introduction

**Figure 1:**
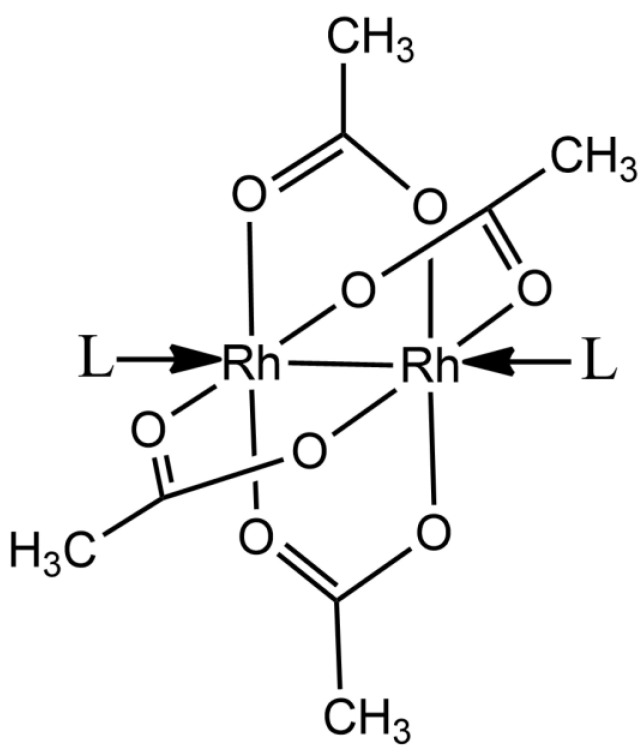
Paddle-wheel structure of [Rh_2_(µ-O_2_CCH_3_)_4_]. L indicates possible ligands at the axial position.

Dirhodium(II) tetracarboxylates [Rh_2_(O_2_CR)_4_]L_2_ (R = CH_3_^−^, CH_3_CH_2_^−^, etc.; L = a possible donor ligand) are efficient catalysts for the decomposition of diazo compounds [1], nitrene transfer reaction [2], carbene insertion into aliphatic and aromatic C–H bonds [3,4], aromatic cycloaddition [5], hydrosilylation of alkynes [6,7], and oxidation of alcohols [8]. The most-studied member of this class of metal compounds, the paddlewheel dirhodium tetraacetate complex ([Rh_2_(μ-O_2_CCH_3_)_4_]) (Figure 1), has also been considered as a promising catalyst for photochemical hydrogen evolution [9] and as a potential anticancer agent [10,11]. Indeed, [Rh_2_(μ-O_2_CCH_3_)_4_] and its derivatives are cytotoxic in vivo for Ehrlich ascites, L1210 tumors, sarcoma 180, and P388 leukemia [12,13,14,15,16,17,18]. Furthermore, it can be used to produce artificial metalloenzymes for selective olefin cyclopropanation [19,20].

Due to their possible role in the definition of the mechanism of action of these potential anticancer agents, the interactions of [Rh_2_(μ-O_2_CCH_3_)_4_] with amino acids, peptides, and proteins have been studied, but contrasting results have been reported in the literature [21,22,23,24,25,26,27,28,29,30]. Reaction of [Rh_2_(μ-O_2_CCH_3_)_4_] with Cys and its derivatives and with glutathione leads to oxidation of the Rh(II)–Rh(II) dimeric unit and formation of Rh(III)–Rh(III)-containing dimeric and oligomeric species [21]. Asp or Glu side chains of some peptides have been demonstrated to be involved in the formation of Rh/peptide adducts via acetate replacement [23,24]. Binding to human serum albumin (HSA) and other free SH-containing enzymes occurs via oxidation of the Rh(II)–Rh(II) dimeric unit, metal compound decomposition, and binding of monometallic Rh(III) centers to proteins [24]. Other authors reported that dirhodium carboxylates bind His residues of HSA [30]. Displacement of acetate ligands and coordination of Cys residues to the dirhodium center has been reported by Stillman and coworkers who studied the reaction of [Rh_2_(μ-O_2_CCH_3_)_4_] with metallothionin [29].

In this frame, the exact definition, at atomic level, of the structures of the reaction products of dirhodium compounds with peptides and proteins is important for a deeper understanding of the dirhodium binding to biologically important molecules. Structural studies are desirable also for the rational design of artificial metalloenzymes based on the incorporation of dirhodium compounds within protein scaffolds. Indeed, [Rh_2_(O_2_CR)_4_]L_2_ has been conjugated to peptides and proteins producing biohybrid materials with interesting catalytic activities [19,20,31].

Although the structural studies on Rh/protein adducts are important, scarce information on this topic is reported in literature, determining a substantial information gap.

Recently, the high-resolution structure of the [Rh_2_(μ-O_2_CCH_3_)_4_]/bovine pancreatic ribonuclease (RNase A) adduct formed upon soaking of protein crystals in solutions containing [Rh_2_(μ-O_2_CCH_3_)_4_] has been reported [32]. Data indicate that the dirhodium compound binds the side chains of two His residues at the axial coordination site, retaining the acetate ligands, without affecting the overall structure of the enzyme. This contrasts with what happens when the analogous paddlewheel diruthenium tetraacetate compound (Ru_2_(μ-O_2_CCH_3_)_4_]) reacts with the model protein hen egg white lysozyme (HEWL) [33].

To obtain further insights into the interaction of dirhodium compounds with proteins and to directly compare the behavior of [Rh_2_(μ-O_2_CCH_3_)_4_] in its reaction with HEWL with that of the analogous diruthenium compound [Ru_2_(μ-O_2_CCH_3_)_4_] [33], we solved the crystal structures of the adducts formed in the reaction of HEWL with [Rh_2_(μ-O_2_CCH_3_)_4_] under different experimental conditions, including that used to determine the structure of the [Ru_2_(μ-O_2_CCH_3_)_4_]/HEWL adduct, using soaking and cocrystallization procedures. HEWL is extensively used as model protein for metalation studies [33,34,35,36,37,38,39,40,41,42,43,44] and as a biocompatible carrier for metal compounds [45,46,47]. The structures of adducts formed upon reaction of [Ru_2_(μ-O_2_CCH_3_)_4_] with this protein provide insightful data into the protein binding capabilities of dirhodium tetraacetate complexes. The structures here reported have been also compared with those obtained when crystals of HEWL have been treated with RhCl_3_ [34] and with the structure of the [Rh_2_(μ-O_2_CCH_3_)_4_]/RNase A adduct [32].

## 2. Results and Discussion

### 2.1. Structures of the Rh/HEWL Adducts Obtained by Cocrystallization

The structure of the adduct formed upon reaction of [Rh_2_(μ-O_2_CCH_3_)_4_] with HEWL were investigated by X-ray crystallography. Six complete X-ray diffraction datasets were collected (Table 1)—four datasets were collected on crystals of HEWL grown after incubation of the protein in the presence of [Rh_2_(μ-O_2_CCH_3_)_4_] and two datasets were collected using crystals of Rh/HEWL adducts obtained upon exposure of metal-free HEWL crystals to solutions containing [Rh_2_(μ-O_2_CCH_3_)_4_]. Crystals of the adducts obtained by cocrystallization procedure were grown under the same crystallization conditions (see Methods for details). Thus, as expected, these structures were rather similar. The aim of the replicated structural determination was to provide additional observations for the electron density maps of the Rh binding sites. In these structures the Rh binding did not affect the overall features of HEWL. CA root mean square deviations from the structure of the native protein (Protein Data Bank (PDB) code 193L) were within the range 0.14 to 0.26 Å. Analysis of the difference Fourier Fo-Fc electron density maps suggests that the dirhodium compound breaks down in the reaction with the protein and that Rh atoms bind HEWL with a partial (<1.0) occupancy. Due to partial occupancy and disorder, in some cases Rh ligands have not been modelled.

**Figure 2:**
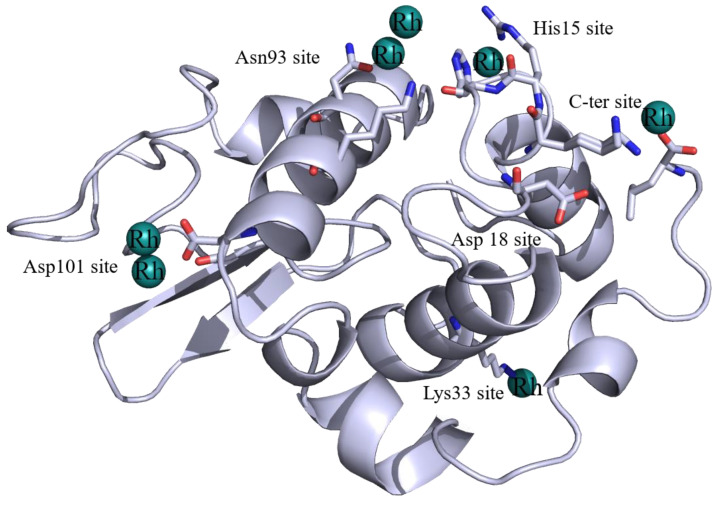
Overall structure of HEWL with identification of Rh binding sites.

**Figure 3:**
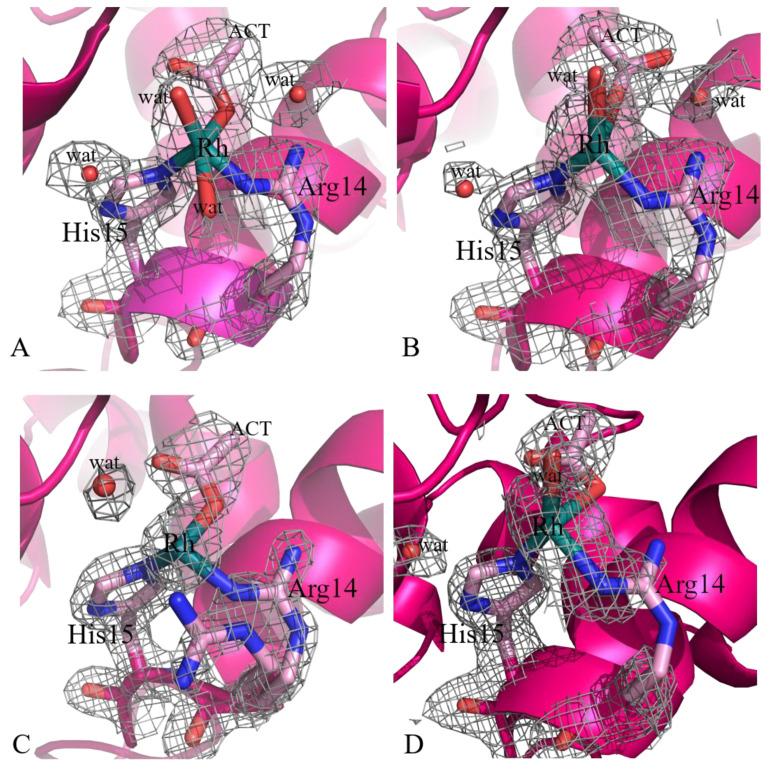
Details of the Rh binding site close to Arg14 and His15 in the crystals 1–4 (panels **A**–**D**, respectively). 2Fo-Fc electron density maps are contoured at 1.0 σ (grey). An example of anomalous difference electron density map of this site is reported in Supporting Information (Figure S1).

A summary of the Rh binding sites and Rh ligands identified in the four structures are reported in Table 2; Rh binding sites are mapped on the HEWL structure in Figure 2. Common to these structures is the presence of a Rh center bound to the side chains of His15 and Arg14 (Figure 3).

**Figure 4:**
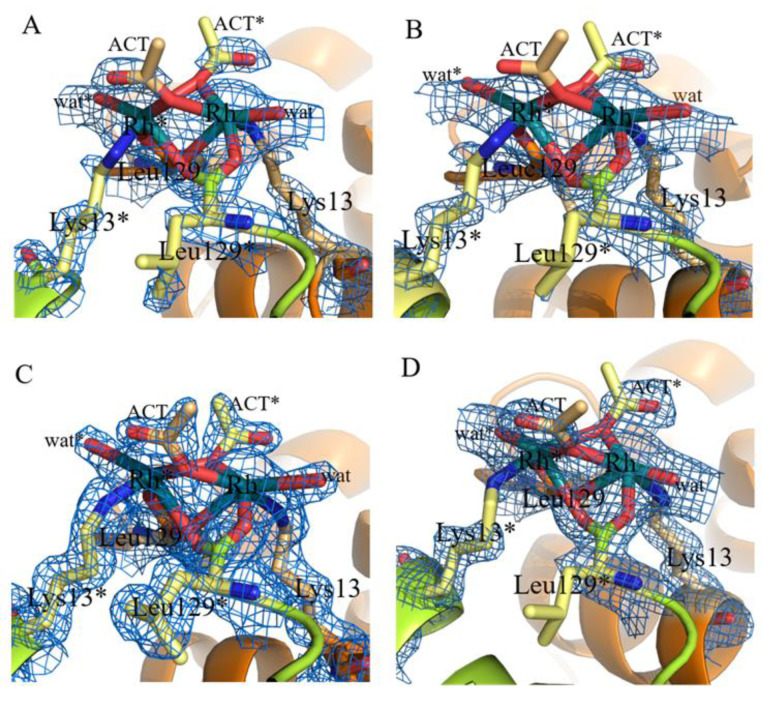
Details of Rh binding sites close to C-terminal tail and the side chain of Lys13 in the Crystals 1–4 (**A**–**D**). 2Fo-Fc electron density maps are contoured at 0.7 σ (cyan). An example of anomalous difference electron density map of this site is reported in Supporting Information (Figure S1).

His15 is a known metal binding site of HEWL [35,36,37,38,39,40,41,42,43,44]. In analogy with what was observed in the binding of Pt [35,36], Ru [37,38,39,40,41], Au [42,43], and Ag [44], the Rh center coordinates the imidazole rings of the His side chain at NE2 atom. The Rh–NE2 distance is on average equal to 2.35 Å. This bond length is within the usual range of Rh–N bond lengths (in small molecules Rh–N distances range from 2.224 Å (refcode: CUCROY) to 2.357 Å (refcode: VUGFOT) [48]. Besides His and Arg side chains, Rh is also bound to an O atom of an acetate (ACT in Figure 3) and to one or two water molecules. The sixth ligand completing the distorted octahedral metal geometry is not observed in the electron density map. Rh–O bond lengths (1.91–2.67 Å) are in some cases significantly larger than expected (Rh–O distance is 2.0–2.2 Å in the structures of small molecules). Occupancy of Rh at this binding site ranges from 0.30 to 0.50, indicating a good degree of protein metalation. Additional Rh centers are found close to the binary axis, at the protein/protein interface (Figure 4). At this binding site, two strong blobs of electron density are found in both Fourier difference and anomalous difference electron density maps at a distance of 3.22–3.42 Å. Here, Rh atoms are bound to the C-terminal carboxylates of two symmetry-related molecules. Besides the C-terminal tails, close to the metal centers, there are the NZ atoms of the side chain of Lys13, acetate ions, and water molecules. In some structures the metal ligands are not well defined. Refined occupancy values (from 0.65 to 0.70) indicate that the two Rh centers are not alternative to each other, demonstrating the existence of a Rh–Rh dimeric unit, and suggest that this could be the main Rh binding site. The distance between the two Rh centers is within the range of typical dimeric Rh(III)–Rh(III) unit distances. In fact, literature data [49] indicate that the reaction of sodium thiolates with dirhodium tetraacetate breaks up the Rh_2_(O_2_CCH_3_)_4_ framework in the presence of O_2_ to form an oligomeric chain of triply S-bridged Rh(III) ions, each with six Rh–S (2.36 ± 0.02 Å) bonds. In this structure, the Rh(III) distance is 3.18 ± 0.02 Å. This distance is similar to that previously found for the aerobic reaction product from aqueous solutions of Rh_2_(O_2_CCH_3_)_4_ and glutathione and in agreement with EXAFS data [22,49]. Thus, it seems that in our structure the acetate and the carboxylate C-terminal tails of the two symmetry-related molecules can bridge two metal centers acting as the sulfur atoms in the Rh(III)–S–Rh(III) unit found in the product of the reaction with sodium thiolate [49]. Since HEWL does not contain free thiolates, other agents should be involved in the oxidation of the metal.

**Figure 5:**
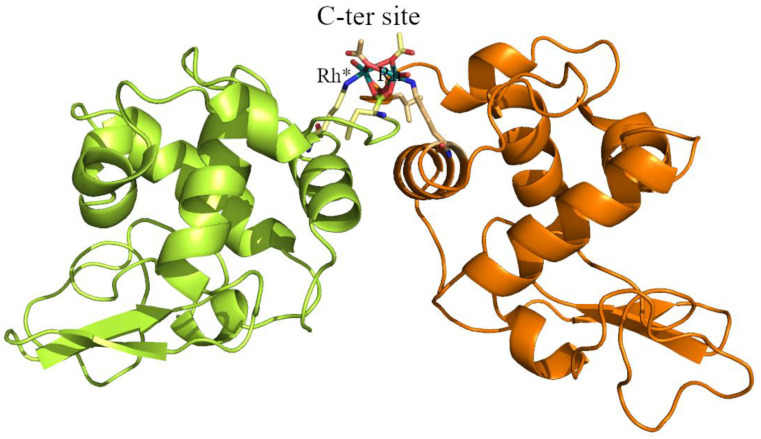
Structure of the cross-linked hen egg white lysozyme (HEWL) dimer formed in the Rh/HEWL crystals obtained when the protein is treated with [Rh_2_(μ-O_2_CCH_3_)_4_].* Referes to the symmetry related molecule.

**Figure 6:**
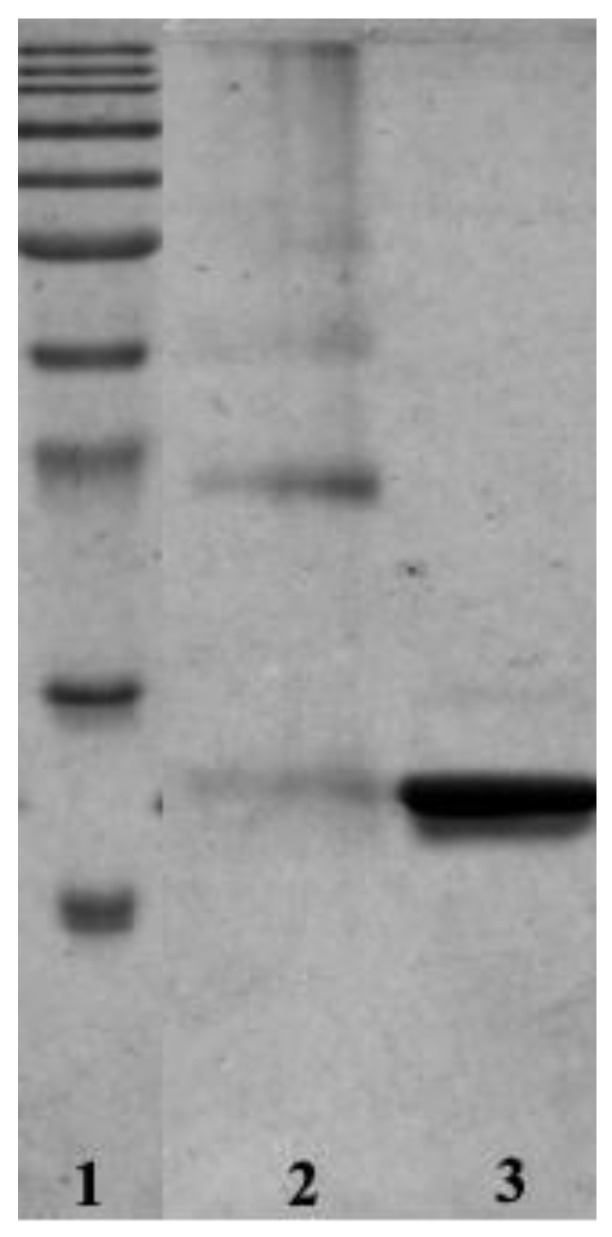
SDS-PAGE analysis of dissolved Rh/HEWL crystals. Line 1, markers; Line 2, dissolved Rh/HEWL crystals; and Line 3, dissolved metal-free HEWL crystals.

The position of the metal centers at the protein/protein interface suggests an experiment that can be used to verify the existence of the suggested model. The two Rh centers are found at the interface between two HEWL chains, acting as cross-linkers. To verify that cross-linked HEWL dimers (Figure 5) are formed as a result of the interaction of the Rh(III)–Rh(III) unit with protein residues, crystals of the adduct obtained in the reaction of HEWL with dirhodium tetraacetate have been dissolved and subjected to SDS-PAGE (Figure 6). This experiment indicates the formation of SDS-resistant protein dimers. The existence of HEWL dimers in crystals of HEWL grown in the presence of [Rh_2_(μ-O_2_CCH_3_)_4_] and their absence in crystals of metal-free HEWL indirectly validate our structural model.

The finding that the dirhodium compound can act as protein cross-linking agent suggests that it can induce the formation of protein dimers also in the cellular milieu.

The C-terminal tail has been previously found to be involved in the recognition of metal centers in the structure of the Ru/HEWL adducts formed upon reaction of the protein with CORM-2 (Ru_2_Cl_4_(CO)_6_) solved by Ueno and coworkers [38] and in the structure of the HEWL adduct with a Re compound solved by Helliwell and coworkers [50]. In the former structure, two Ru centers are close to each other, as in the Rh/HEWL adduct here determined, but the two Ru centers have occupancy < 0.5 (0.30 and 0.50, respectively). In the latter, Re atoms (occupancy 0.39 and 0.55) are bound to the carboxylate oxygens with a Re–O distance of 2.1 and 2.7 Å [50].

**Figure 7:**
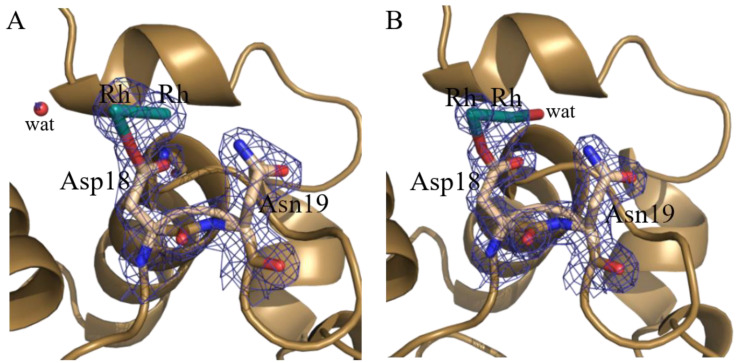
Details of Rh binding sites close to the side chain of Asp18 in the Crystals 1 and 2 ((**A**,**B**), respectively). 2Fo-Fc electron density maps are contoured at 1.0 σ (blue). An example of anomalous difference electron density map of this site is reported in Supporting Information (Figure S1).

Major differences between the four structures obtained by cocrystallization are located close to the side chain of Asp18, where two Rh atoms with low occupancy (0.20/0.20 and 0.40/0.40) are observed in two out of the four obtained structures (Crystals 1 and 2) (Figure 7). Although we cannot exclude that a single Rh atom could occupy two alternate positions at this site, it seems plausible that a dirhodium center can be bound to the protein, considering that the two Rh atoms are at a distance that is compatible with the presence of a Rh(II)–Rh(II) center (2.31 and 2.48 Å) and have the same occupancy and very similar B-factors (B-factor differences are 7.7% and 10.6% in the two structures). In this frame it should be recalled that Rh–Rh distances in small molecules range from 2.388 Å (refcode; GERQUP) to 2.431 Å (refcode: FUNDEX01). In both structures derived from Crystals 1 and 2, the low occupancy of the metallic fragment(s) prevents the modelling of Rh ligands at this site.

Interestingly, Asp18 has been already found to be involved in the interaction with metal centers in the structure of other adducts with metal compounds [38,39,50,51,52].

### 2.2. Structures of the Rh/HEWL Adduct Obtained by Soaking Procedure

Additional information on possible Rh binding sites on HEWL surface was obtained by analyzing the structures of the Rh/HEWL adducts obtained using the soaking strategy. Crystal structure of the Rh/HEWL adduct formed within HEWL crystals grown under the same conditions used to grow those of the adducts obtained by cocrystallization is very similar to those previously described (root mean square deviations (rmsd) = 0.19–0.24 Å). Inspection of this structure revealed that Rh is bound to the side chains of Arg14 and His15 and to the C-terminal carboxylate and the side chain of Lys13 (see Supporting Information Figure S2). This finding, once again [51], indicates that in the reaction between protein and metallodrugs, soaking and the cocrystallization procedure very often provide similar results.

**Figure 8:**
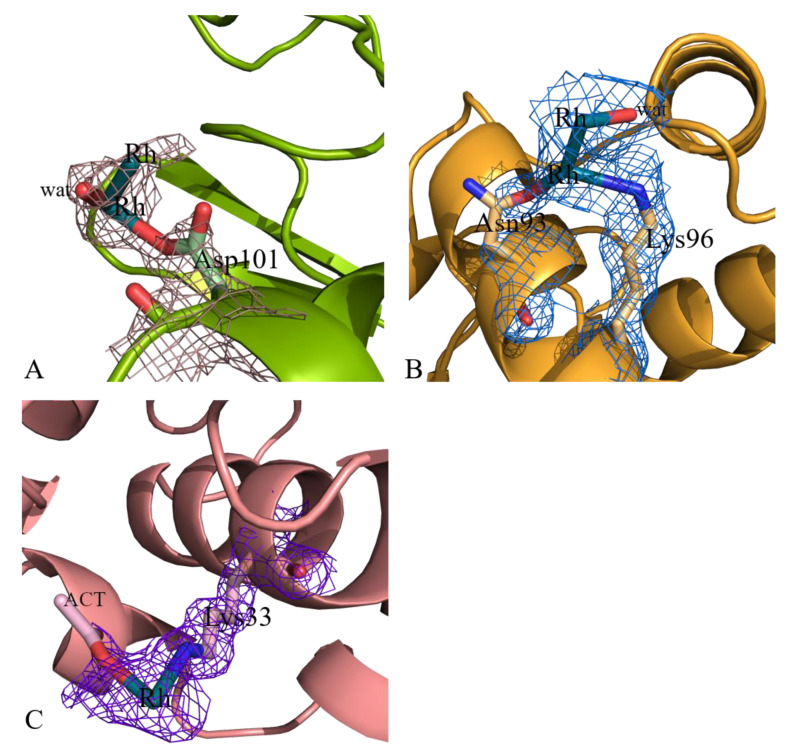
Details of the Rh binding sites close to side chains of Asp101 (**A**), Asn93 and Lys96 (**B**) and Lys33 (**C**) in Crystal 6. 2Fo-Fc electron density maps are contoured at 1.0 σ.

On the contrary, the structure of the adduct formed within HEWL crystals grown using 10 mM HEPES pH 7.5 and 2.00 M sodium formate as reservoir showed unexpected and interesting results. In this structure, beyond the Rh centers at level of Arg14/His15, Lys13 and C-terminal carboxylate (see Supporting Information Figure S3), three additional Rh binding sites were found. In particular, Rh centers were observed close to side chains of Asp101, Asn93 and Lys96, and Lys33 (Figure 8). Rmsd of this structure from those previously described is within the range 0.19–0.27 Å indicating that differences in the Rh binding sites are not associated with a large variation of the overall structure of the protein.

Close to the side chain of Asp101 two Rh atoms at a distance of 2.29 Å (occupancy = 0.40/0.40) were observed (Figure 8A). The metals are at distances of 2.32–2.56 Å from the oxygens of the Asp side chain. As discussed below for the Asp18 binding site, considering the Rh–Rh distance and the metal occupancies it is plausible that a dirhodium center is bound to the protein at this site, although the possibility of the existence of a Rh atom occupying alternative positions close to the side chain of the Asp cannot be excluded.

Close to the side chains of Asn93 and Lys96, another two Rh atoms (occupancy = 0.30/0.30) are found (Figure 8B). In this site, the Rh–Rh distance is 2.48 Å. One of the two metal centers is at distances of 2.42 Å and 2.80 Å from the two side chains. Since the other Rh is not bound to protein atoms, the possibility that at this site a single Rh atom occupying alternate positions can be present seems to be very unlikely, supporting the idea that a dirhodium center is here bound to the protein.

At the last binding site, close to the side chains of Lys33, a monometallic fragment is found. At this site, an acetate ion is bound to the Rh center (Figure 8C).

These results indicate that the reactivity of the investigated dirhodium compound with HEWL strongly depends on the conditions used for the reaction of the compound with this biological macromolecule and that monometallic and dimetallic fragments coexist under the same experimental conditions.

### 2.3. Comparison with Literature Data

The six structures of the Rh/HEWL adducts here reported were first compared with the structure of the adduct obtained when HEWL reacts with [Ru_2_(μ-O_2_CCH_3_)_4_] [33] and with those found when the protein was incubated in the presence of RhCl_3_ under different experimental conditions [34]. The overall conformation of HEWL is conserved in these structures. Rmsd of CA atoms was within the range 0.17–0.23 Å.

At variance with what was found in many binding sites observed in the structures of the adducts of HEWL with [Rh_2_(μ-O_2_CCH_3_)_4_] here reported, in the structure of the [Ru_2_(μ-O_2_CCH_3_)_4_]/HEWL adduct, the diruthenium center is conserved:the Ru–Ru center binds the side chains of Asp101 and Asp119 [33] and the acetate ligands remain attached to the dimetallic centers. Thus, although diruthenium and dirhodium compounds have similar structures they present a significantly different reactivity with the same protein under the same experimental conditions.

The Rh binding sites observed in our structures are also different from those found when the protein reacts with RhCl_3_ [34]. Common sites are the side chains of Arg14/His15 and Asp18. In one of the structures of the HEWL adducts formed with RhCl_3_, the Rh center that is coordinated to Asp18 is also in contact with the side chain of Asn19 [34]. Significant differences have been observed at the other binding sites. In the structures reported by Watanabe and coworkers [34], Rh centers are bound to the side chains of Asn46/Asp52, Asn65, Asp87, and Asp119. These binding sites were not found in our structures. These findings indicate that Rh oxidation state and Rh ligands play a significant role in determining the final metal binding sites, suggesting that different Rh ligands can be used to drive the metal compounds towards different protein binding sites or even towards different molecular targets.

Rh/HEWL structures here reported have been also compared with that of the adduct formed upon reaction of RNase A with the dirhodium compound [32]. In the latter structure, the whole [Rh_2_(μ-O_2_CCH_3_)_4_] coordinates the side chain of His residues at the axial coordination site [32]. This demonstrates that [Rh_2_(μ-O_2_CCH_3_)_4_] can react with different proteins producing different products. Furthermore, data suggest that in the reaction with proteins dirhodium carboxylates can retain their structure or can be degraded and that both monometallic and dimetallic fragments can bind the protein. Data also indicate that His side chain can coordinate both the whole [Rh_2_(μ-O_2_CCH_3_)_4_] compound or Rh-containing fragments obtained by degradation of the original compound.

## 3. Materials and Methods

### 3.1. Preparation, Characterization in Solution and Crystallization of the Adduct Formed Upon Reaction of [Rh_2_(μ-O_2_CCH_3_)_4_] with HEWL

[Rh_2_(μ-O_2_CCH_3_)_4_] and HEWL were purchased from Sigma Chemical Co (Merck Life Science S.r.l., Milan, Italy) at highest grade of purity and used without further purification. Crystals of the Rh/HEWL adduct that was formed upon reaction of [Rh_2_(μ-O_2_CCH_3_)_4_] with HEWL in 10:1 metallodrug to protein molar ratio (incubation time is 24 h) were grown by the hanging drop vapor diffusion method mixing 1 μL of adduct solution (protein concentration 14 mg mL^−1^) with an equal volume of a solution containing 20% ethylene glycol, 0.100 M sodium acetate at pH 4.5, and 0.600 M sodium nitrate.

Crystals of Rh/HEWL adducts were also obtained using the soaking strategy on native crystals of HEWL grown in 20% ethylene glycol, 0.100 M sodium acetate at pH 4.5, and 0.600 M sodium nitrate or in 0.010 M HEPES pH 7.5 and 2.00 M sodium formate. Crystals of metal-free HEWL were soaked in a solution of the reservoir containing a large excess of [Rh_2_(μ-O_2_CCH_3_)_4_] for 1 year and 7 days, respectively.

Before the crystallization trials it was verified that HEWL retained its secondary structure content in the presence of the metal compound by collecting circular dichroism (CD) spectra of the protein incubated for 24 h in the presence of [Rh_2_(μ-O_2_CCH_3_)_4_] at different protein to metal ratios (Figure S4). It was also verified that the compound was stable under the conditions used to crystallize the protein by collecting UV–Vis absorption spectra of [Rh_2_(μ-O_2_CCH_3_)_4_] over time (Figure S5).

Circular dichroism (CD) experiments were collected using a Jasco J-810 spectropolarimeter (JASCO Corp., Milan, Italy) at 25 °C. Quartz cells with path length of 0.1 cm were used in the far-UV region from 200 to 250 nm. Three scans were registered for each spectrum; contributions from the corresponding references were subtracted, and the signal converted to mean residue ellipticity in units of deg/cm^2^/dmol. Spectra were acquired in 0.004 M HEPES at pH 7.5 and in 0.004 M sodium acetate buffer at pH 4.5. Other experimental settings were: 50 nm/min scan speed, 2.0 nm band width, 0.2 nm resolution, 50 mdeg sensitivity, and 4 s response.

A Jasco UV–Vis spectrophotometer was used to collect absorption spectra of [Rh_2_(μ-O_2_CCH_3_)_4_]. In particular, spectra of the dirhodium compound were registered in 0.010 M sodium acetate pH 4.5 or 0.010 M HEPES at pH 7.5 using 0.008 M [Rh_2_(μ-O_2_CCH_3_)_4_]. UV–Vis spectra were acquired over time at room temperature in the 340–700 nm range every 1 nm at a scan rate of 400 nm/min.

### 3.2. Data Collection and Refinement

X-ray diffraction data collection was carried out on six different crystals at 100K. Four crystals of the adduct (Crystals 1–4) were obtained by cocrystallization in 20% ethylene glycol, 0.100 M sodium acetate at pH 4.5, and 0.600 M sodium nitrate. Crystals 5–6 were obtained by the soaking procedure, as described above. Crystals 1 and 2 were flash frozen without cryoprotectant. Crystals 3–6 were flash-frozen with 30% (*v*/*v*) glycerol as a cryo-protectant. The first two data collections were carried out at the CNR Institute of Biostructure and Bioimages in Naples, Italy, using a Saturn944 CCD detector equipped with CuKα X-ray radiation from a Rigaku Micromax 007 HF generator. Data collection for Crystals 3 and 6 was carried out at the XRD2 beamline of Elettra synchrotron in Trieste, Italy. Diffraction data for Crystals 4 and 5 were collected at Diamond Light Source synchrotron in Oxfordshire, UK. Data were scaled using HKL2000 [53], Mosflm/Scala [54] or Autoproc [55]. Data collection and refinement statistics are reported in Table 1.

Phaser [56] was used to solve the structures with the molecular replacement method; PDB code 193L [57] was used as a starting model. Restrained refinement was performed using Refmac5 [58]. Model building was carried out using Coot [59]. Besides Rh atoms and water molecules, nitrate and acetate ions were added to the structures. Nitrate ions were located in positions corresponding to those of other nitrate ions in structures of HEWL reported in the PDB. Acetate ions were placed in the remaining trigonal planar electron density maps when proper interactions with protein residues were formed. The presence of Rh centers was verified by inspection of anomalous difference maps. Rh occupancy was evaluated minimizing the peaks in correspondence of the Rh center in the 2Fo-Fc electron density maps.

Structures were validated using the PDB validation server (www.rcsb.org) and Coot routines [59]. Figures were generated using Pymol (www.pymol.org). The PDB codes for the six structures are 7bdz, 7be0, 7be1, 7beb, 7bec, and 7be2.

## 4. Conclusions

In solution studies demonstrated that dirhodium carboxylate compounds are able to bind proteins [12,22,23,26,28,29]. However, there are only very scarce structural information on this interaction. Here, we have refined six single crystal structures of adducts formed upon reaction of [Rh_2_(μ-O_2_CCH_3_)_4_] with the model protein HEWL, obtained under three different experimental conditions. The work provides rare examples of structures of adducts formed in the reaction of a protein with a Rh compound. The structures indicate that the metal compound in part degrades in the crystals. The anchoring of Rh-containing fragments to the protein does not affect HEWL overall conformation. Multiple Rh binding sites exist on the protein structure, characterized by different metal occupancy factors, which could be associated with different affinities of the Rh compound for the numerous binding sites. Monometallic Rh fragments bind the side chains of Arg14 and His15, and the side chain of Lys33 with occupancy equal to 0.25–0.50 and 0.20, respectively, while possible dimetallic Rh(II)–Rh(II) units are found close to carboxylates of Asp18 and of Asp101, and between the side chains of Asn93 and Lys96 with low occupancy (occupancy factor = 0.30 on average). A dirhodium unit with a Rh–Rh distance of 3.2–3.4 Å, which suggests oxidation of the metal centers, is bound to the C-terminal carboxylate and to the side chain of Lys13 at the interface between two symmetry-related molecules. At the latter binding site, an unusual structural motif has been identified, with acetate ions and the C-terminal tail that act as bridging ligands. As judged from occupancy factor of Rh centers (and considering that Rh is found at this site in all the structures of Rh/protein adducts here refined) this should be the main binding site.

These data demonstrate that metal centers experience different environments and thus they could have a distinct reactivity. Thus, Rh/HEWL adducts and even crystals of Rh/HEWL adducts here characterized could be used as catalysts. The significance of different binding sites for the anticancer activity of [Rh_2_(μ-O_2_CCH_3_)_4_] is not obvious, but our data suggest that it is possible that in the cellular milieu the Rh compound could degrade upon reaction with proteins and could induce the formation of protein dimers and other aggregates. This could alter the activity of the Rh-bound enzymes and could be potentially dangerous for the organism. Structural data also suggest that, given its high reactivity, this compound could have different molecular targets. The different Rh binding sites could perform distinct functions and/or biological roles.

The Rh binding sites observed in our structures are distinct from those obtained in the reaction of the protein with RhCl_3_ [34]. This finding suggests that different Rh ligands can be used to drive Rh compounds towards different protein binding sites and different molecular targets. Thus, fine tuning of Rh ligands could improve the performances of these potential drugs.

Comparison of the present results with previous literature structural data obtained in the reaction of [Ru_2_(μ-O_2_CCH_3_)_4_] with HEWL [33] and of [Rh_2_(μ-O_2_CCH_3_)_4_] with RNase A [32] indicates that [Rh_2_(μ-O_2_CCH_3_)_4_] reacts with the hen egg enzyme differently from the analogous diruthenium compound [33] and that the dirhodium carboxylates can react with proteins in different ways, both retaining the dirhodium center or upon degradation ([32] and present results). Data also show that free cysteines are not the only residues responsible for dirhodium carboxylate decomposition. These results suggest that upon synthesis of artificial metalloenzymes based on dirhodium carboxylate compounds, the products of the reaction between [Rh_2_(μ-O_2_CCH_3_)_4_] and proteins should be carefully analyzed from the structural point of view, since unexpected features could be identified.

## Figures and Tables

**Table 1:** Data collection and refinement statistics.

	Crystal 1	Crystal 2	Crystal 3	Crystal 4	Crystal 5	Crystal 6
Procedure	Cocrystallization	Cocrystallization	Cocrystallization	Cocrystallization	Soaking	Soaking
Crystallization conditions	20% ethylene glycol, 0.100 M sodium acetate at pH 4.5, and 0.6 M sodium nitrate					
Data collection						
Space group	P4_3_2_1_2	P4_3_2_1_2	P4_3_2_1_2	P4_3_2_1_2	P4_3_2_1_2	P4_3_2_1_2
a = b (Å)	77.964	77.948	78.360	78.604	78.007	78.930
c (Å)	37.291	37.380	37.610	37.307	37.245	37.120
Resolution range (Å)	25.48–1.94	26.99–1.62	78.36–1.40	55.58–1.32	35.19–1.74	55.81–1.65
	(1.97–1.94)	(1.65–1.62)	(1.47–1.40)	(1.37–1.32)	(1.83–1.74)	(1.80–1.65)
Unique reflections	8895 (426)	15,018 (623)	22,922 (3274)	26,513 (1327)	11,348 (567)	11,800 (591)
Completeness (%)	99.0 (97.1)	98.7 (86.6)	100 (100)	95.4 (51.1)	91.1 (36.0)	94.0 (52.6)
Redundancy	5.3 (3.7)	9.0 (1.9)	19.6 (16.6)	25.8 (25.9)	25.0 (27.6)	24.5 (22.4)
† Rmerge (%)	0.095 (0.440)	0.068 (0.190)	0.099 (2.00)	0.047 (2.55)	0.070 (3.12)	0.120 (3.23)
Rpim	0.045 (0.249)	0.021 (0.152)	0.023 (0.519)	0.013 (0.720)	0.020 (0.848)	0.025 (0.680)
Average I/σ(I)	14.9 (2.2)	56.7 (3.4)	10.2 (1.0)	28.8 (1.1)	24.7 (1.1)	22.5 (1.3)
CC_1/2_	0.997 (0.808)	0.999 (0.942)	0.999 (0.701)	1.00 (0.612)	1.00 (0.474)	1.00 (0.51)
Anomalous completeness (%)	99.1 (94.1)	96.8 (67.3)	100 (100)	95.3 (52.1)	90.8 (36.6)	93.8 (51.4)
Anom. Redundancy	2.9 (2.0)	5.0 (1.3)	10.3 (8.5)	13.8 (13.4)	13.7 (14.5)	13.4 (12.0)
Refinement						
Resolution range (Å)	25.48–1.94	26.99–1.62	55.41–1.40	55.58–1.32	35.19–1.74	55.81–1.65
Anom. Redundancy	2.9 (2.0)	5.0 (1.3)	10.3 (8.5)	13.8 (13.4)	13.7 (14.5)	13.4 (12.0)
Refinement						
Resolution range (Å)	25.48–1.94	26.99–1.62	55.41–1.40	55.58–1.32	35.19–1.74	55.81–1.65
Number of reflections (working set)	8445	14,241	22,453	25,227	10,794	11,205
Number of reflections (test set)	8445	14,241	22,453	25,227	10,794	11,205
R-factor/R-free (%)	15.6/21.7	16.4/20.2	19.0/20.9	17.7/22.3	18.5/22.8	18.1/23.4
N. of atoms	1221	1231	1283	1275	1129	1139
Average B-factors (Å^2^)						
All atoms	21.1	21.4	16.8	27.3	42.9	30.6
Rh occupancy	0.70/0.35/0.40/0.40	0.65/0.50/0.20/0.20	0.65/0.30	0.70/0.30	0.25/0.40/0.20	0.40/0.45/0.35/0.20/0.30/0.30/0.40
Rh atoms	31.7/33.7/67.9/75.3	30.8/46.9/39.3/42.5	23.1/26.7	35.5/41.7	81.1/73.9/65.2	59.6/69.0/66.9/49.3/68.7/57.4/79.8
Root mean square deviations						
Bond lengths (Å)	0.009	0.013	0.013	0.013	0.008	0.008
Bond angles (°)	2.38	1.96	1.87	2.39	1.55	1.55
Ramachandran statistics (Coot analysis)						
N. of residues in						
Allowed/disallowed regions	4/0	4/0	3/0	3/0	4/0	3/0

† Rmerge = Σ_h_Σ_i_ |I(h,i) − <I(h)>|/Σ_h_Σ_i_ I(h,i), where I(h,i) is the intensity of the ith measurement of reflection h and <I(h)> is the mean value of the intensity of reflection h.

**Table 2:** Rh binding sites found in the six structures of Rh/HEWL adducts obtained upon reaction of the protein with [Rh_2_(μ-O_2_CCH_3_)_4_]. Table describes the ligands at each Rh binding site. Values in parentheses refer to the occupancy of metal and ligands.* refers to atoms from symmetry related molecules.

	Crystal 1	Crystal 2	Rh/HEWL Adduct Ligands Crystal 3	Crystal 4	Crystal 5	Crystal 6
**His15 site**	Rh (0.35) His15 (1) Arg14 (1) Act (1) H_2_O (0.35) H_2_O (0.65)	Rh (0.5) His15 (1) Arg14 (1) Act (1) H_2_O (0.5)	Rh (0.3) His15 (1) Arg14 (0.5) Act (1)	Rh (0.3) His15 (1) Arg14 (0.5) Act (1) H_2_O (0.3) H_2_O (0.3)	Rh (0.25) His15 (1) Arg14 (0.5) Act (1) H_2_O (1)	Rh (0.35) His15 (1) Arg14 (1) H_2_O (0.35) H_2_O (0.35)
**Asp18 site**	Rh (0.40) Rh (0.40) Asp18 (1)	Rh (0.20) Rh (0.20) Asp18 (1) H_2_O (0.20)				
**C-ter site**	Rh (0.65)	Rh (0.65)	Rh (0.65)	Rh (0.7)	Rh (0.4) Rh * (0.4) Leu129 (1) Leu129 * (1) Lys13 (1) Lys13 * (1)	Rh (0.45) Rh * (0.45) Leu129 (1) Leu129 * (1) Lys13 (1) Lys13 * (1) H_2_O (0.5) H_2_O * (0.5)
	Rh * (0.65)	Rh * (0.65)	Rh * (0.65)	Rh* (0.7)		
	Leu129 (1)	Leu129 (1)	Leu129 (1)	Leu129 (1)		
	Leu129 * (1)	Leu129 * (1)	Leu129 * (1)	Leu129 * (1)		
	Lys13 (1)	Lys13 (1)	Lys13 (1)	Lys13 (1)		
	Lys13 * (1)	Lys13 * (1)	Lys13 * (1)	Lys13 * (1)		
	Act (0.60)	Act (0.65)	Act (0.65)	Act (0.7)		
	Act * (0.60)	Act * (0.65)	Act * (0.65)	Act * (0.7)		
	H_2_O (0.65)	H_2_O (0.65)	H_2_O (0.65)	H_2_O (0.7)		
	H_2_O * (0.65)	H_2_O * (0.65)	H_2_O * (0.65)	H_2_O * (0.7)		
**Asp101 site**						Rh (0.40)
						Rh (0.40)
						Asp101 (1)
						H_2_O (0.40)
**Lys33 site**						Rh (0.20)
						Lys33 (1)
						Act (0.33)
**Asn93 site**						Rh (0.30)
						Rh (0.30)
						Asn93 (1)
						Lys96 (1)
						H_2_O (1)

## Data Availability

Data available in a publicly accessible repository.

## Supplementary Materials

Supplementary materials can be found at https://www.mdpi.com/1422-0067/22/3/1496/s1.

## Abbreviations

CD	Circular dichroism
HEWL	Hen egg white lysozyme
HSA	Human serum albumin
PDB	Protein Data Bank
Rmsd	Root mean square deviation
RNase A	Bovine pancreatic ribonuclease
SDS	Sodium dodecyl sulfate
SDS-PAGE	Sodium dodecyl sulfate-polyacrylamide gel electrophoresis
UV	Ultraviolet
UV–Vis	Ultraviolet–visible
