# Supplementary material for: Unusual Structural Features in the Adduct of Dirhodium Tetraacetate with Lysozyme
Source: Int J Mol Sci. 2021 Feb 2;22(3):1496. doi: 10.3390/ijms22031496 (PMC7867343; doi:10.3390/ijms22031496)
Supplement: Supplementary file 1 [file ijms-22-01496-s001.pdf]

Article

Unusual Structural Features in the Adduct of Dirhodium Tetraacetate with Lysozyme

Domenico Loreto [1], Giarita Ferraro [2] and Antonello Merlino [1,*]

[1] Department of Chemical Sciences, University of Naples Federico II, 80126 Napoli, Italy; domenico.loreto@unina.it
[2] Department of Chemistry "Ugo Schiff", University of Florence, via della Lastruccia, 3-13, 50019 Sesto Fiorentino, Florence, Italy; giarita.ferraro@unifi.it
* Correspondence: antonello.merlino@unina.it

Supporting information

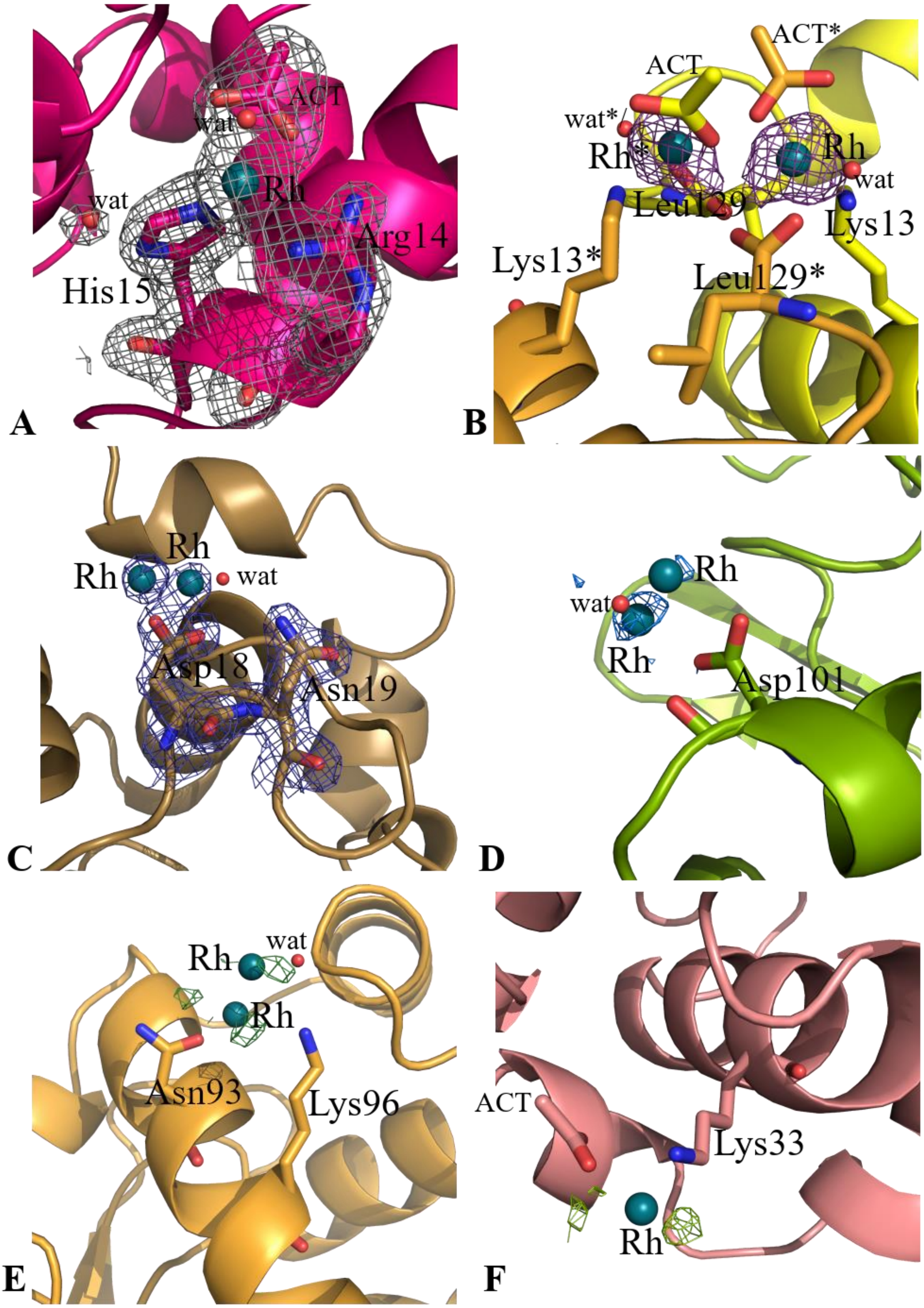

Figure S1. Examples of anomalous difference electron density maps at the Rh binding sites close to side chains of Arg14 and His15 (**A**), close to the C-terminal tail (**B**), close to side chain of Asp18 (**C**), Asp101 (**D**), Asn93 and Lys96 (**E**) and of Lys33 (**F**) in the structures of Rh/HEWL adducts here reported. Maps are contoured at 3.0 σ in panels A and B and at 2.0 σ in the other panels.

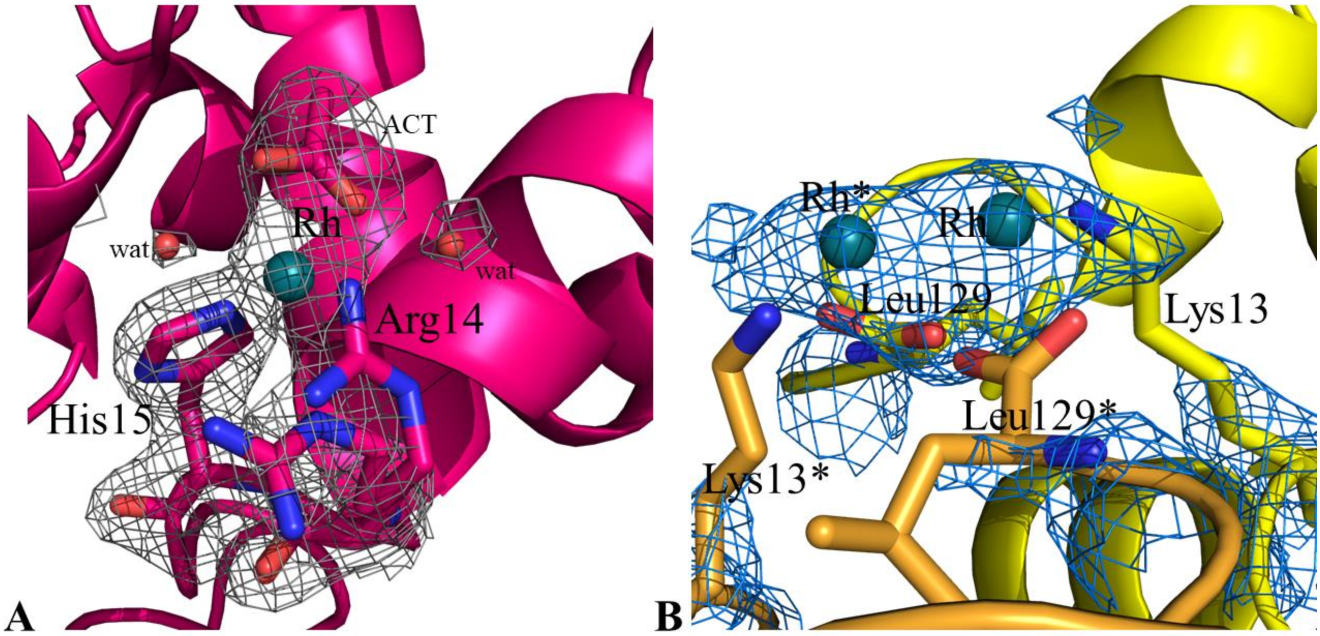

Figure S2. Details of Rh binding sites close to the side chains of Arg14 and His15 (**A**) and close to the C-terminal tail and the side chain of Lys13 (**B**) in Crystal 5. 2Fo-Fc electron density maps are contoured at 1.0 σ. * refers to atoms from symmetry related molecules.

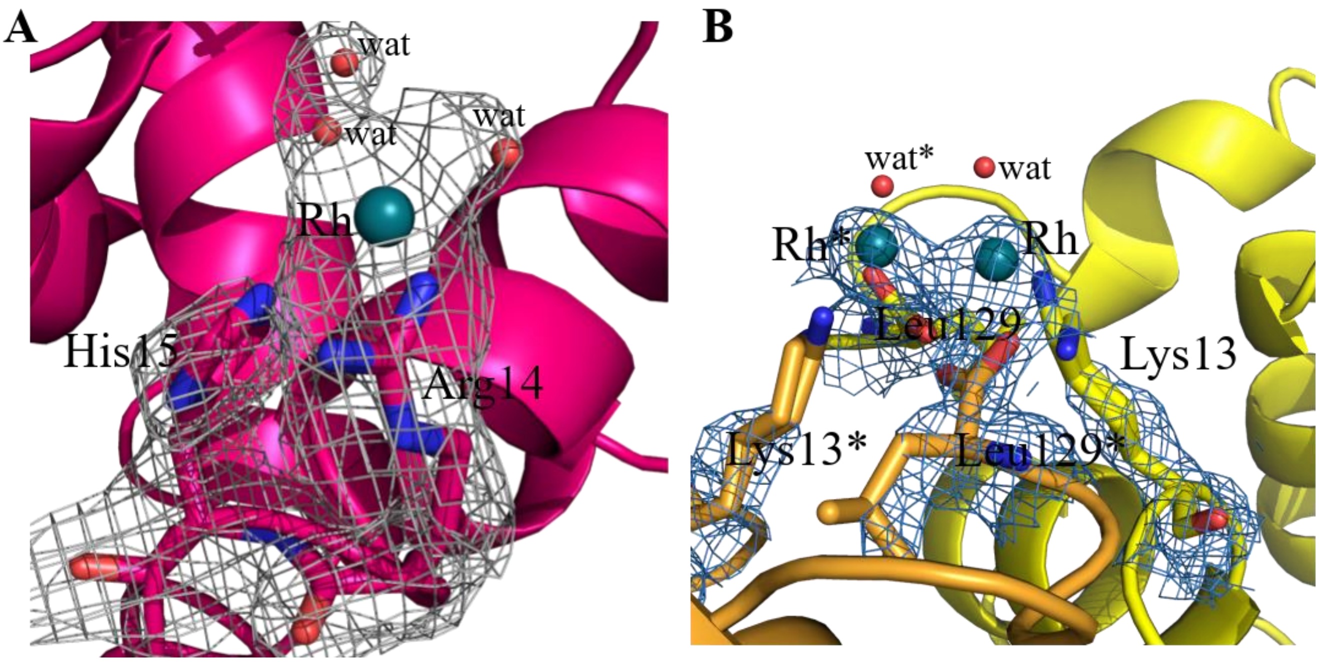

Figure S3. Details of Rh binding sites close to the side chains of Arg14 and His15 (**A**) and close to the C-terminal tail and the side chain of Lys13 (**B**) in Crystal 6. 2Fo-Fc electron density maps are contoured at 1.0 σ.

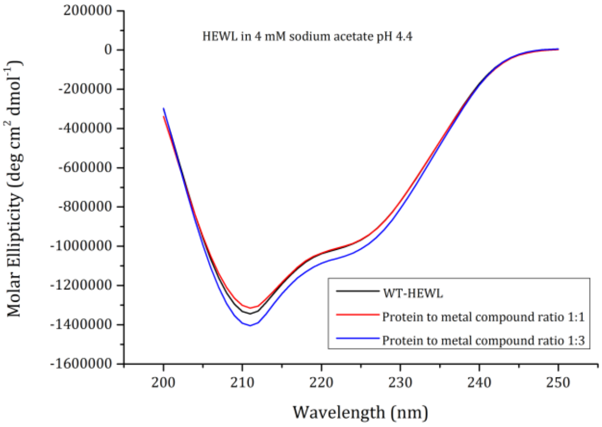

A

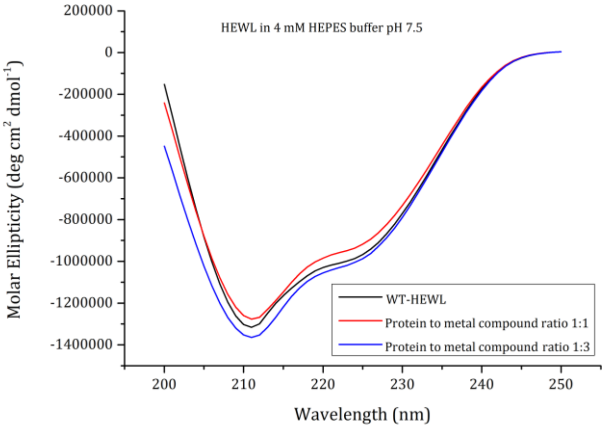

B

Figure S4. CD spectra of HEWL incubated for 24 h in the presence of $[Rh_2(\mu\text{-}O_2CCH_3)_4]$ at different protein to metal compound ratios in (**A**) 4 mM sodium acetate buffer pH 4.4 and (**B**) 4 mM HEPES buffer pH 7.5.

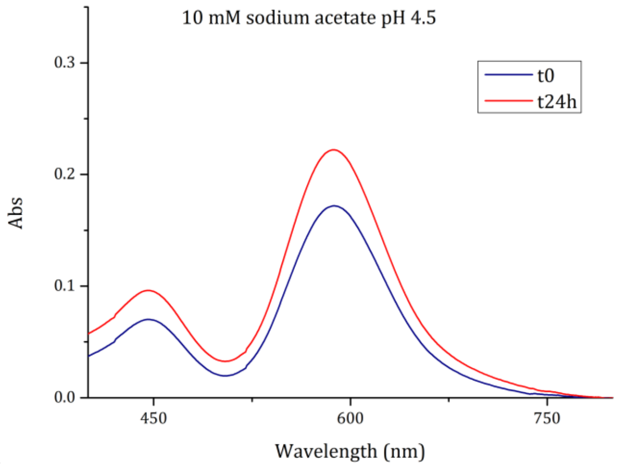

A

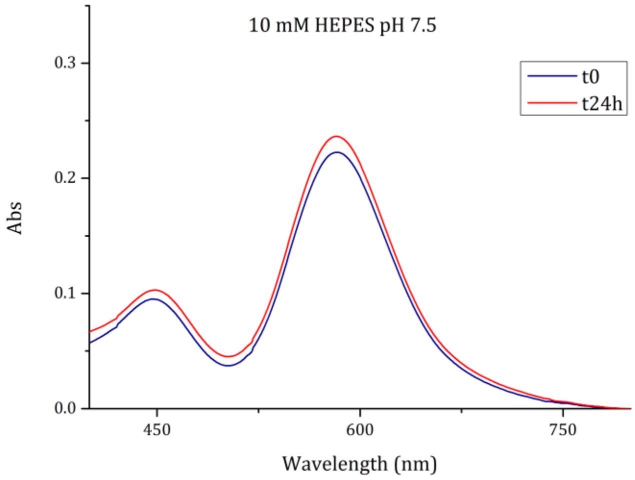

B

Figure S5. UV-Vis spectra of [$Rh_2(\mu\text{-}O_2CCH_3)_4$] in (**A**) 10 mM sodium acetate buffer at pH 4.5 and (**B**) 10 mM HEPES pH 7.5.
